# Anti-malarial effect of novel chloroquine derivatives as agents for the treatment of malaria

**DOI:** 10.1186/s12936-017-1725-z

**Published:** 2017-02-17

**Authors:** Seon-Ju Yeo, Dong-Xu Liu, Hak Sung Kim, Hyun Park

**Affiliations:** 1 Department of Infection Biology, School of Medicine, Zoonosis Research Center, Wonkwang University, 460, Iksan-daero, Iksan, Jeolabuk-do 54538 Republic of Korea; 2 College of Pharmacy, Institute of Pharmaceutical Research and Development, Wonkwang University, 460, Iksan-daero, Iksan, Jeolabuk-do 54538 Republic of Korea

**Keywords:** Chloroquine derivatives, SKM13, Anti-malarial efficacy, α,β-unsaturated amides

## Abstract

**Background:**

The widespread emergence of anti-malarial drug resistance has necessitated the discovery of novel anti-malarial drug candidates. In this study, chloroquine derivatives were evaluated for the improved anti-malarial activity.

**Results:**

Novel two derivatives (SKM13 and SKM14) were synthesized based on the chloroquine (CQ) template containing modified side chains such as α,β-unsaturated amides and phenylmethyl group. The selective index indicated that SKM13 was 1.28-fold more effective than CQ against the CQ-resistant strain *Plasmodium falciparum*. An in vivo mouse study demonstrated that SKM13 (20 mg/kg) could completely inhibit *Plasmodium berghei* growth in blood and increased the survival rate from 40 to 100% at 12 days after infection. Haematological parameters [red blood cell (RBC) count, haemoglobin level, and haematocrit level] were observed as an indication of clinical malarial anaemia during an evaluation of the efficacy of SKM13 in a 4-day suppression test. An in vivo study showed a decrease of greater than 70% in the number of RBC in *P. berghei*-infected mice over 12 days, but the SKM13 (20 mg/kg)-treated group showed no loss of RBC.

**Conclusions:**

CQ derivatives with substituents such as α,β-unsaturated amides and phenylmethyl group have enhanced anti-malarial activity against the CQ-resistant strain *P. falciparum*, and SKM13 is an excellent anti-malarial drug candidate in mice model.

**Electronic supplementary material:**

The online version of this article (doi:10.1186/s12936-017-1725-z) contains supplementary material, which is available to authorized users.

## Background

Malaria is a disease affecting humans, caused by a protozoan parasite of the *Plasmodium* genus, with 107 countries and territories having areas at risk of transmission [1]. The World Health Organization (WHO) reported the occurrence of 214 million cases worldwide in 2015, and the death of 438,000 people, mostly children in the African region [2].

Successful malaria control in the past decade was dependent on treatment with efficacious anti-malarial drugs [3]. Quinoline drugs such as chloroquine (CQ) and quinine were the cornerstone of malaria treatment [4]. Quinine was used as anti-malarial medication from the seventeenth century until the 1920s, when CQ, a more effective synthetic anti-malarial, became available [5]. However, the extensive use of CQ led to the development of a chloroquine-resistant malaria parasite *Plasmodium falciparum* in Southeast Asia, Oceania, and South America in the late 1950s and early 1960s [6]. This drug resistance inspired a significant effort throughout the twentieth century to identify new anti-malarial agents for the improvement of global public health. There is still a need to produce “novel” drugs with different properties, which has led to dramatic changes in the way new targets are identified [7].

Although the molecular basis of chloroquine action is yet to be properly elucidated, the mechanism has traditionally been considered to occur through interference in the haemozoin crystal formation of the *Plasmodium* species, leading to detoxification of the malaria parasite [8, 9]. Chloroquine-resistant *P. falciparum* survives by reduction of drug accumulation in the digestive vacuole. In addition to being effective as an anti-malarial medication, CQ has emerged as a prospective adjunct with antiviral effects [10, 11], antitumour activity [12], and as an effector of cell-death by altering lysosomal function [13].

More efficacious drugs are currently available [5]. For example, artemisinin has been reported as a potent anti-malarial drug, but the emergence of resistance has increased the failure rate of artemisinin-based combination therapy [14–16]. As resistance to existing drugs develops, new drugs need to be introduced; for *P. falciparum,* the use of a combination of several drugs with different modes of action is recommended to provide an adequate cure rate and delay the development of resistance.

Several novel drug candidates based on the CQ structure, with modifications of both the side chain and the quinoline ring, have been reported [17–19]. In a previous study, the Michael-acceptor role of the α,β-unsaturated amide, which mimics the functional group present in gallinamide A and many anti-malarial chalcones, was found to stabilize the thiolate covalent bond between calpain, a cysteine protease required for cell cycle progression in *Plasmodium* parasites [20], and the β carbon of the α,β-unsaturated amide [21, 22].

In the present study, two novel derivatives were designed based on the CQ structural template with a modified side chain, such as α,β-unsaturated amides and phenylmethyl group. These two derivatives were evaluated for anti-malarial activity in vitro and in vivo.

## Methods

### Reagents

Chloroquine and atovaquone were purchased from Sigma Aldrich (St. Louis, USA). SYTOX® Green nucleic acid stain was purchased from Life Technologies (Carlsbad, USA). The CellTiter 96® AQueous One Solution reagent was purchased from Promega (Madison, USA).

### Synthesis of SKM13 and SKM14

SKM13 and SKM14 were synthesized using the following scheme: (1) the coupling of 4,7-dichloroquinoline and phenylalanine [23]; (2) the formation of Weinreb amide [24]; (3) reduction to aldehyde [25]; (4) Horner–Wadsworth–Emmons reaction with the amide phosphonate [26]. The chemical structures were confirmed by proton NMR.

### In vitro culture of *Plasmodium* species

*Plasmodium falciparum* 3D7 (American Type Culture Collection, ATCC PRA-405D) and *P. falciparum* FCR3 (American Type Culture Collection, ATCC® 30932) were purchased from the ATCC (Manassas, USA). The chloroquine-susceptible strain *Plasmodium berghei* NK65 (MRA-268) and the atovaquone-resistant strain *P. berghei* NAT (MRA-415) were purchased from Bei Resources (Manassas, USA). The *P. falciparum* strains 3D7 and FCR3 were grown in human erythrocytes as previously described [27]. Briefly, parasites were maintained in continuous culture with 5% haematocrit of type O human red blood cells suspended in Roswell Park Memorial Institute (RPMI) 1640 medium supplemented with 24 mM NaHCO_3_, 25 mM HEPES, 0.8% hypoxanthine, 0.9% Albumax, and 25 μg/mL of gentamicin. The 6-well plates were placed in an incubator (atmosphere: 5% CO_2_, 5% O_2_, and 90% N_2_) at 37 °C and the medium was changed daily when the level of parasitaemia was at least 5%. The parasite density was determined by Giemsa staining of thin smears and expressed as a percentage of infected erythrocytes in a field of a total of 500 erythrocytes.

### In vitro anti-malarial activity by FACS analysis

To assess the effect of the compounds on malarial parasite growth, the parasites were seeded in 48-well plates at a density of 0.5 in 2% haematocrit. The CQ compounds were then serially diluted in medium and incubated with the parasites for 48 h without any medium change. Finally, 100 μL of the *P. falciparum* cultures were withdrawn from each well of the 48-well plate and 1 μL of the blood pellet was mixed in 5 mM SYTOX green solution to obtain a final volume of 1.5 mL. The mixture was left to stand in the dark for 30 min at room temperature and the anti-malarial activity of the compounds was analysed by fluorescence-activated cell sorting (FACS) using a FACS Calibur flow cytometer (BD Biosciences, Franklin Lakes, USA).

### In vitro cytotoxicity

The MDCK cells were purchased from the Korean Cell Line Bank. The cells were seeded in a 96-well plate in Dulbecco’s modified Eagle’s medium (DMEM) supplemented with 10% fetal bovine serum (FBS) and incubated with serially diluted CQ compounds for 48 h.

After the incubation period, 20 μL of CellTiter 96® AQueous One Solution reagent was added to each well. After incubation for 1 h at 37 °C in a humidified 5% CO_2_ atmosphere, the absorbance was measured at 490 nm using an enzyme-linked immunosorbent assay (ELISA) plate reader.

### Test for suppressive activity (Peter’s 4-day test)

ICR female mice (Orient Bio Co., Seongnam, South Korea), 7 weeks old, were housed at Wonkwang University. All mice were bred and maintained under constant conditions. Animal experiments were performed using an experimental protocol approved by the Animal Care and Use Committee at Wonkwang University (WKU16-40).

The atovaquone-resistant strain *P. berghei* NAT and the chloroquine-sensitive strain *P. berghei* NK65 were used to set up an infected mouse model. The parasites kept in liquid nitrogen were thawed at 37 °C and maintained by the serial passage of blood from mouse to mouse. In this study, each mouse was injected with 10^7^ *P. berghei*-infected erythrocytes/100 μL by intraperitoneal (i.p.) injection. At one day post-infection (dpi), drug treatment with SKM13 (20 mg/kg) was commenced. Atovaquone (4 mg/kg) and chloroquine (10 mg/kg) were intravenously (i.v.) administered once a day for four consecutive days as the control drug. Each day, tail blood was collected for FACS assessment of parasitaemia. Mouse weight and survival was checked every day, and haematology assays were conducted at days 3, 6, 9, and 12 dpi.

### Haematological assay

The haematological testing was conducted using a fully automated blood cell counter LC-660 (HORIBA Medical, Seoul, South Korea). The parameters evaluated included white blood cell count, red blood cell count, haemoglobin, and haematocrit.

### Statistical analysis

Results were statistically analysed using GraphPad Prism software 5.0. The experiments were compared using one-way ANOVA followed by Bonferroni’s multiple comparison test. All results are expressed as mean ± SD (standard deviation of the mean). *P* values of less than 0.05 were considered statistically significant.

## Results

### Characterization of SKM13 and SKM14

**Fig. 1:**
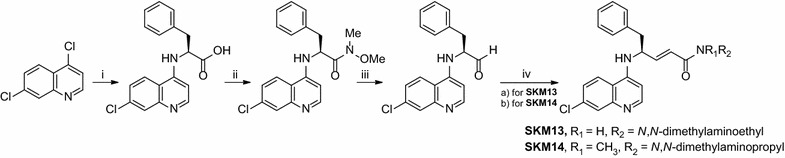
Synthesis of SKM13 and SKM14. Reaction conditions: *i* l-phenylalanine, phenol, 140 °C, 1 h, 55%. *ii N*,*O*-dimethylhydroxylamine·HCl, EDAC·HCl, DMAP, TEA, DCM, rt, overnight, 72%. *iii* DIBAL, −40 °C, DCM, 5 h, 95%. *iv* phosphonate, DCM, K^*t*^OBu, 0 °C; (*a*) for **SKM13** (71%), diethyl (2-((2-(dimethylamino)ethyl)(methyl)amino)-2-oxoethyl)phosphonate; (*b*) for **SKM14** (70%), diethyl (2-((3-(dimethylamino)propyl)amino)-2-oxoethyl)phosphonate

Two novel CQ derivatives (SKM13 and SKM14) were synthesized in this study **(**Fig. 1). CQ and two CQ derivatives have two differences of chemical structure. In the structure of CQ, there is a small methyl group on the side chain [28] but two CQ derivatives have phenylmethyl group derived from phenylalanine at the same position. Besides, both CQ derivatives have α,β-unsaturated amide instead of methyl group of CQ. A little difference of chemical structure between SKM13 and SKM14 indicates that the α,β-unsaturated amide in SKM13 is shorter than that in SKM14.

### In vitro anti-malarial activity

After 48-h exposure to drugs, the standard in vitro assays for anti-parasitic effects were determined by FACS analysis. It showed that SKM13 and SKM14 had higher IC_50_ values than CQ; IC_50_ values of 0.014 ± 0.002 (mean ± SD) μM, 0.17 ± 0.01 and 0.23 ± 0.01 μM for CQ, SKM13, and SKM14, respectively, against CQ-susceptible *P. falciparum* (3D7) (Table 1), In MDCK cells, the CC_50_ value indicated that SKM13 and SKM14 were more cytotoxic than CQ. Finally, it turned out that SKM13 and SKM14 had much lower selective index (SI) value (CC_50_/IC_50_) than that CQ. In contrast, the IC_50_ values for CQ-resistant *P. falciparum* (FCR3) confirmed the higher efficacy of SKM13 than CQ: CQ, SKM13, and SKM14 had IC_50_ values of 0.62 ± 0.04 (mean ± SD) μM, 0.37 ± 0.01 and 0.59 ± 0.02 μM, respectively. Therefore, the IC_50_ values of SKM13 and SKM14 were 0.6- and 0.84-fold lower than that of CQ against CQ-resistant *P. falciparum* (FCR3), indicating that CQ derivatives were more effective than CQ as anti-malarial. Finally, the SI values showed that SKM13 was 1.28-fold more effective than CQ against CQ-resistant *P. falciparum*. In contrast, SI value of SKM14 was lower than that of CQ. FACS analysis results can be found in the Additional file 1: Figures S1–S6).

**Table 1:** In vitro anti-malarial activity

Strain	Compound	(IC_50_, μM)	Relative IC_50_	(CC_50_, μM)	Selective index(SI) (CC_50_/IC_50_)	Relative SI
3D7	Chloroquine	0.014 ± 0.002	1.00	121.90 ± 8.51	8707.10	1.00
	SKM13	0.17 ± 0.01	12.14	93.11 ± 5.56	547.70	0.06
	SKM14	0.23 ± 0.01	16.42	47.06 ± 3.21	204.16	0.02
FCR3	Chloroquine	0.62 ± 0.04	1.00	121.90 ± 8.51	196.60	1.00
	SKM13	0.37 ± 0.01	0.60	93.11 ± 5.36	251.70	1.28
	SKM14	0.52 ± 0.02	0.84	47.06 ± 3.21	90.50	0.46

Effects of chloroquine derivatives on the proliferation of CQ-susceptible and resistant strains of *P. falciparum*

Chloroquine-susceptible (3D7) and -resistant (FCR3) *P. falciparum* strains were cultured in the presence of increasing concentrations of CQ or each of the CQ derivatives. The values shown are the mean ± standard deviation (SD) from three independent experiments performed in triplicate

### Evaluation of the suppressive activity of SKM13 (Peter’s 4-day test)

**Fig. 2:**
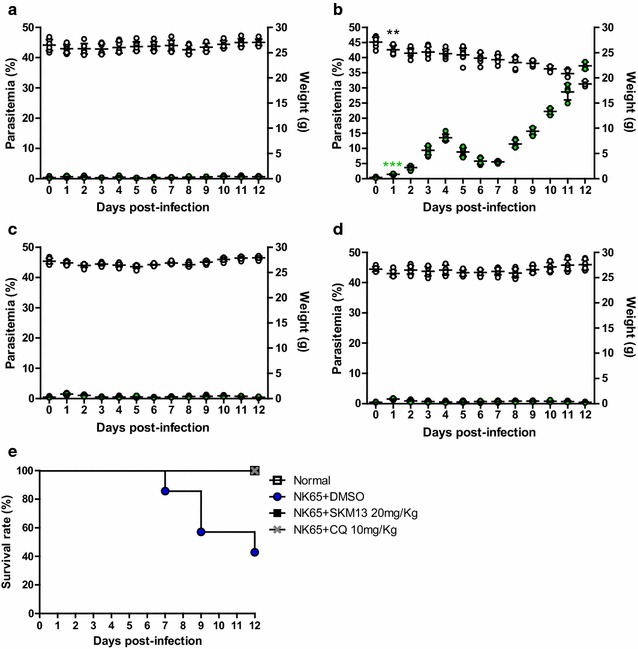
Curative effect of SKM13 against chloroquine-susceptible strain *P. berghei* in a 4-day suppression test. *Plasmodium berghei* (NK65) parasites (10^7^/mouse) were injected via the intraperitoneal (i.p.) route in mice (*n* = 7). After 1 day, drugs were administered via mouse vein for four consecutive days. After drug treatment, parasitaemia and weight were measured every day (**a** normal group, **b** *P. berghei*-infected group, **c** SKM13-treated group, **d** CQ-treated group; *green circle*, parasitaemia; *open circle*, body weight) (**P < 0.01, ***P < 0.001). The values shown are the mean ± SD. The survival rate (% of control) is shown in (**e**)

The anti-malarial activity of SKM13 in a rodent model was assessed by a 4-day suppression test of 20 mg/kg of SKM13 in mice. As seen in Fig. 2, CQ-susceptible *P. berghei* NK65 parasites (10^**7**^/mouse) were i.p. injected into ICR mice. After one day, SKM13 (20 mg/kg, in doses of 10 mg/kg, twice daily) and CQ (10 mg/kg, once daily) were i.v. administered for four consecutive days.

In the parasite-infected mice, a rise in parasitaemia was observed at 2 dpi, which decreased until 7 dpi. After 8 dpi, parasitaemia increased exponentially and high parasitaemia (about 30%) was observed until 12 dpi **(**Fig. 2b). Parasitaemia in the SKM13-treated group (Fig. 2c) was significantly suppressed compared to that reported for the parasite-infected group (*P* < 0.05), and the CQ-treated group (Fig. 2d) showed no parasitaemia.

Body weight decreased in the parasite-infected mice. Normal body weight (27.09 ± 1.01 g, mean ± SD) significantly decreased (25.58 ± 0.93 g) at 2 dpi (*P* < 0.01), but remained unchanged in the SKM13-treated group (26.96 ± 0.56 g) at 12 dpi, demonstrating that SKM13 (20 mg/kg) was effective in the maintenance of normal mouse metabolism until 12 dpi. CQ (10 mg/kg) also maintained normal mouse body weight.

Analysis of survival rate showed that SKM13 was effective in treating *P. berghei* infection. The parasite-infected group showed 20% mortality at 7 dpi, 40% mortality at 9 dpi, and 60% mortality at 12 dpi. SKM13 treatment resulted in 100% survival rate in mice at 12 dpi (Fig. 2e).

To assess the anti-malarial activity of SKM13 against drug-resistant *P. berghei*, atovaquone-resistant *P. berghei* NAT parasites (10^7^/mouse) were i.p. injected into ICR mice. After 1 day, SKM13 (20 mg/kg, in doses of 10 mg/kg, twice daily), atovaquone (4 mg/kg, once daily), and CQ (10 mg/kg, once daily), were separately i.v. administered for 4 consecutive days.

**Fig. 3:**
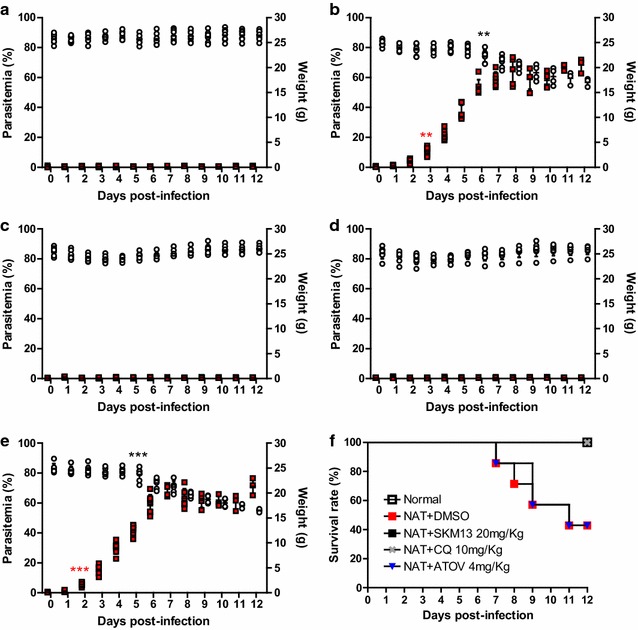
Effect of SKM13 against chloroquine-resistant strain *P. berghei* in a 4-day suppression test. *Plasmodium berghei* (NAT) parasites (10^7^/mouse) were injected via the i.p. route in mice (*n* = 7) and after 1 day, drugs were administered in the mouse vein for four consecutive days. After drug treatment, parasitaemia and weight were measured every day (**a** normal group, **b** *P. berghei*-infected group, **c** SKM13-treated group, **d** CQ-treated group, **e** atovaquone-treated group; *red square*, parasitaemia; *white circle*, body weight) (**P < 0.01, ***P < 0.001). The values shown are the mean ± SD. The survival rate (% of control) is shown in (**f**)

At 3 dpi, parasitaemia increased in the parasite-infected group and reached a peak at 8 dpi. After 8 dpi, a plateau in the level of parasitaemia was observed until 12 dpi (Fig. 3b). Parasitaemia in the SKM13-treated group (Fig. 3c) and CQ-treated group (Fig. 3d) was significantly suppressed compared to that reported for the parasite group (*P* < 0.05), which showed no parasitaemia. As anticipated, atovaquone (4 mg/kg) was unable to suppress the parasitaemia of atovaquone-resistant *P. berghei* NAT (Fig. 3e).

The normal body weight significantly decreased (*P* < 0.01) at 6 dpi but remained unchanged until 12 dpi in the SKM13-treated group. CQ (10 mg/kg) maintained the body weight of the infected mice. In the atovaquone-resistant *P. berghei* NAT-infected group, the mortality rate at 7 dpi was 20 and 60% at 12 dpi. In contrast, the SKM13-treated group showed 100% survival rate until 12 dpi (Fig. 3f).

### Effect of SKM13 on blood cell parameters

**Fig. 4:**
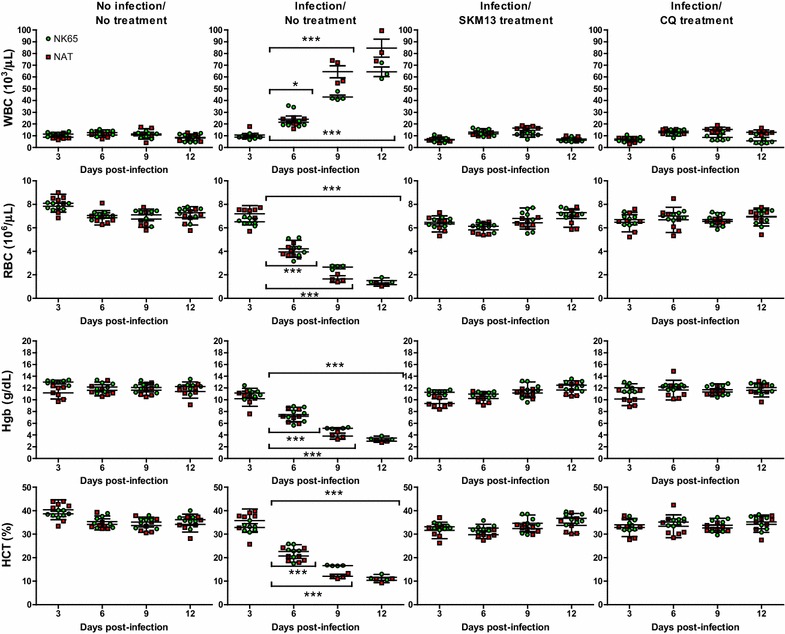
Effect of SKM13 on haematological parameters of *P. berghei*. The effect of SKM13 on haematological indices was examined every 3 days after treatment. At day 6, the WBC count of both of *P. berghei* NK65 and NAT strains significantly increased, and the other three parameters (red blood cell, haemoglobin, and haematocrit) decreased from 3 days after infection. Treatment of SKM13 (20 mg/kg) suppressed the changes of WBC, RBC, haemoglobin, and haematocrit compared with the infected group from 1 to 12 days (*P < 0.05, ***P < 0.0001). The values shown are the mean ± SD

Changes in blood cell count are characteristic of *Plasmodium* infection and haematological changes during the course of a malaria infection, such as anaemia, thrombocytopaenia, leukocytosis, or leucopenia, are well recognized [29]. In this study, WBC counts (mean ± SD) in normal ICR mice were 8.86 ± 1.86 (10^3^/μL), which were in agreement with that reported previously [30]. Both *P. berghei* NK65 and NAT strains significantly increased WBC counts (*P* < 0.05) at 6 dpi and progressively increased WBC counts were observed until 12 dpi (*P* < 0.001). In contrast, SKM13 or CQ treatment did not affect WBC counts (Fig. 4a). In addition to WBC counts, several other factors were observed as major indicators of clinical malaria anaemia in the efficacy evaluation of SKM13. Both *P. berghei* NK65 and NAT strains showed a large decrease in these values, with greater than 70% reduction in the main haematological parameters (RBC, haemoglobin, and haematocrit) for 12 days. However, SKM13 treatment in the 4-day suppression test did not alter the three parameters involved in anaemia (*P* < 0.001) (Fig. 4b–d).

## Discussion

The present study demonstrated that hybrid molecules with a CQ structural template, including a quinolone moiety and a modified side chain such as the α,β-unsaturated amides, could be a useful strategy in the development of anti-malarial drugs.

Because malarial disease is exacerbated by the emergence of drug resistance to efficient treatments, novel anti-malarial medicines are an essential component of malaria control; a continuous search for different drugs with novel chemical structures and mechanism of actions is necessary [31]. As a new strategy in drug design, the hybrid molecule has been characterized by the rational approach of anti-malarial drug design and considered as a method to optimize the therapy with available drugs [32].

One type of these hybrid molecules, quinoline-novel target based hybrid molecules, is widely indicated, because the quinoline entity can inhibit β-haematin formation and contribute to a remarkable drug concentration within the digestive vacuole of *Plasmodium* [33]. So far, a quinoline-based isatin derivative, with the quinoline and isatin groups able to inhibit cysteine proteases, has been reported to show in vitro anti-plasmodial activity [34]. However, the in vivo anti-plasmodial activity of the quinoline-based isatin derivative has not yet been elucidated.

In this study, previous finding that the Michael-acceptor of the α,β-unsaturated amide could form a covalent bond between the thiolate in a cysteine protease and the β carbon of the α,β-unsaturated amide [22] was combined with quinoline as a multi-therapeutic strategy, leading to novel CQ derivatives (SKM13 and SKM14). CQ has a small methyl group on the side chain but SKM13 has a phenylmethyl group derived from phenylalanine. In our result, SKM13 with a bigger substituent than methyl group in CQ showed better inhibitory activity, implicating that it may be preferable to develop derivatives with various larger substituents. Despite the structural similarities of the phenylalanine derivatives SKM13 and SKM14, the selective index of SKM13 was two-fold higher than SKM14. The α,β-unsaturated amide in SKM13 is shorter than that in SKM14. From the results, two effects of the structure of the derivative on the activity of the drug were estimated. First, although the alanine derivative is a similar size to chloroquine, the much larger phenyl group appeared to offer better binding to the target protein. Therefore, the possibility for the use of an amino acid with a larger residue than alanine could be the better strategy for anti-malarial design. Second, as shown by the anti-malarial activity of the derivatives with slightly different alkyl groups located at the nitrogen atom of the amide, the alkyl group could be tuned by size, bulkiness, and polarity.

SKM13 was identified as a potential anti-malarial drug by the complete clearance of parasitaemia, 100% survival rate, and unchanged haematological features in the 4-day suppression test. Specifically, SKM13 was a more efficient anti-malarial candidate than SKM14, implying that α,β-unsaturated amides may be an important strategy for anti-malarial drug design.

 Haematological parameters, such as red blood cells, leukocytes, and thrombocytes, are considered a biomarker of malarial infection and frequently monitored as an indicator of drug efficacy against *Plasmodium* infection. Typically, WBC counts were significantly higher in patients with high parasitaemia than in those with low and moderate parasitaemia [29]. Leukocyte counts, especially of neutrophils, have been reported significantly higher in patients with high parasitaemia compared to that in those with low parasitaemia, which supports the high WBC counts with high parasitaemia in the current rodent model.

Changes in RBC are the most typical feature of malarial infections and the most common complication during malarial infection is anaemia [35]. In the present study, more than 25% loss of total RBC, haemoglobin, and haematocrit demonstrated severe anaemia, and was observed at 6 dpi in both CQ-susceptible and *P. berghei*- (NK65)- and atovaquone-resistant *P. berghei* (NAT)- infected mice. The superior efficacy of SKM13 was supported by the absences of changes in the total RBC, haemoglobin, and haematocrit, which indicated 100% parasite clearance. Therefore, it is considered that SKM13 is one of the compounds that can suppress the potential malarial burden.

Based on an in vitro study, SKM13 may not be as effective as CQ in CQ-susceptible *P. falciparum*, but may be a potentially attractive drug in CQ-resistant *P. falciparum*, which indicates that the mechanism of action may be different between CQ and SKM13.

The limitations of this study included the absence of in vivo results derived from CQ-resistant strain-infected mice. The study of CQ-resistant strain-infected mice will help the interpretation of the efficacy of SKM13. Future elucidation of the mode of action of SKM13 would be necessary for it to be considered as a novel strategy for anti-malarial drug design.

## Conclusions

SKM13 with substituents such as phenylmethyl group and the α,β-unsaturated amides was more effective than CQ against the CQ-resistant strain *P. falciparum*. SKM13 treatment (20 mg/kg) inhibited the *P. berghei* growth completely in the blood of mice and increased the mice survival rate from 40 to 100%. Haematological parameters supported the in vivo efficacy of SKM13 for 4-day suppression test. In conclusion, CQ derivatives with substituents such as the α,β-unsaturated amides and phenylmethyl group can be a useful strategy for improving the anti-malarial activity and SKM13 is an excellent candidate as anti-malarial drug.

## Additional file

**Additional file 1: Figure S1.** FACS analysis for IC_50_ of CQ in *P. falciparum* (3D7). **Figure S2.** FACS analysis for IC_50_ of SKM13 in *P. falciparum* (3D7). **Figure S3.** FACS analysis for IC_50_ of SKM14 in *P. falciparum* (3D7). **Figure S4.** FACS analysis for IC_50_ of CQ in *P. falciparum* (FCR3). **Figure S5.** FACS analysis for IC_50_ of SKM13 in *P. falciparum* (FCR3). **Figure S6.** FACS analysis for IC_50_ of SKM14 in *P. falciparum* (FCR3).
