# Supplementary material for: Anti-malarial effect of novel chloroquine derivatives as agents for the treatment of malaria
Source: Malar J. 2017 Feb 17;16:80. doi: 10.1186/s12936-017-1725-z (PMC5316213; doi:10.1186/s12936-017-1725-z)
Supplement: Supplementary file 1 — Additional file 1: Figure S1. FACS analysis for IC50 of CQ in P. falciparum (3D7). Figure S2. FACS analysis for IC50 of SKM13 in P. falciparum (3D7). Figure S3. FACS analysis for IC50 of SKM14 in P. falciparum (3D7). Figure S4. FACS analysis for IC50 of CQ in P. falciparum (FCR3). Figure S5. FACS analysis for IC50 of SKM13 in P. falciparum (FCR3). Figure S6. FACS analysis for IC50 of SKM14 in P. falciparum (FCR3). [file 12936_2017_1725_MOESM1_ESM.docx]

**Supplementary Information**

**Article title**

Antimalarial effect of (E)-4-((7-chloroquinolin-4-yl)amino)-N-(2-(dimethylamino)ethyl)-5-phenylpent-2-enamide and novel chloroquine derivatives as agents for the treatment of malaria

**Article author**

Seon-Ju Yeo^1^‡, Dong-Xu Liu^1^‡, Hak Sung Kim^2*^, Hyun Park^1*^

^1^Zoonosis Research Center, Department of Infection Biology, School of Medicine, Wonkwang University, Iksan, Jeolabuk-do, 54538, Republic of Korea

^2^College of Pharmacy, Institute of Pharmaceutical Research and Development, Wonkwang University, Iksan, Jeolabuk-do, 54538, Republic of Korea

**Supplementary Figures**

Fig. S1. FACS analysis for IC_50_ of CQ in *P.falciparum* (3D7)

Fig. S2. FACS analysis for IC_50_ of SKM13 in *P.falciparum* (3D7)

Fig. S3. FACS analysis for IC_50_ of SKM14 in *P.falciparum* (3D7)

Fig. S4. FACS analysis for IC_50_ of CQ in *P.falciparum* (FCR3)

Fig. S5. FACS analysis for IC_50_ of SKM13 in *P.falciparum* (FCR3)

Fig. S6. FACS analysis for IC_50_ of SKM14 in *P.falciparum* (FCR3)

**
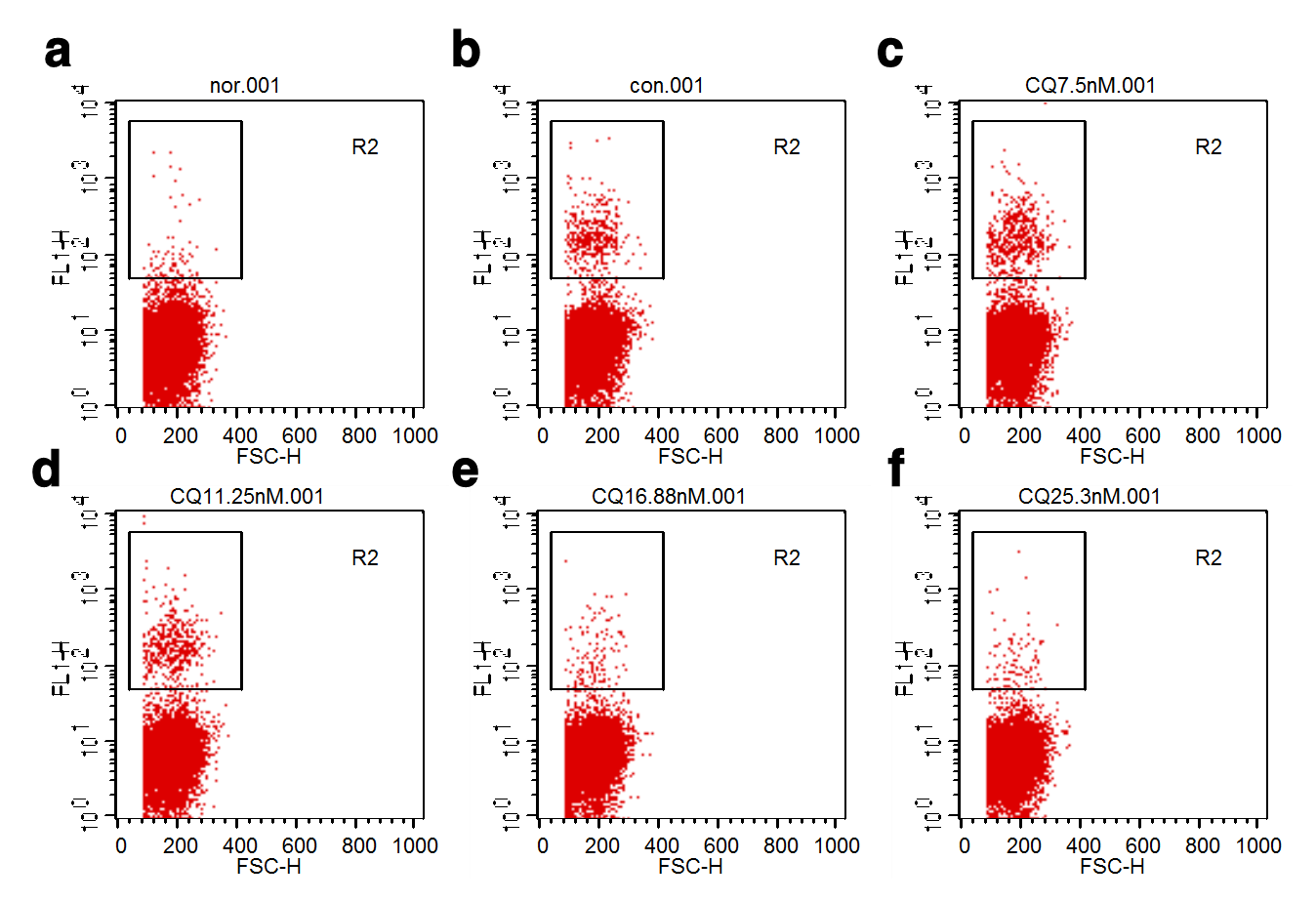
**

**Fig. S1. FACS analysis for IC_50_ of CQ in *P.falciparum* (3D7).** Each small rectangular square indicates the positive fluorescence labeling. **a**, uninfected RBC; **b**, *P.falciparum* (3D7)-infected RBC+DMSO; **c**, infected RBC+ 7.5 nM of CQ; **d**, infected RBC+ 11.25 nM of CQ; **e**, infected RBC+ 16.88 nM of CQ; **f**, infected RBC+ 25.3 nM of CQ.

**
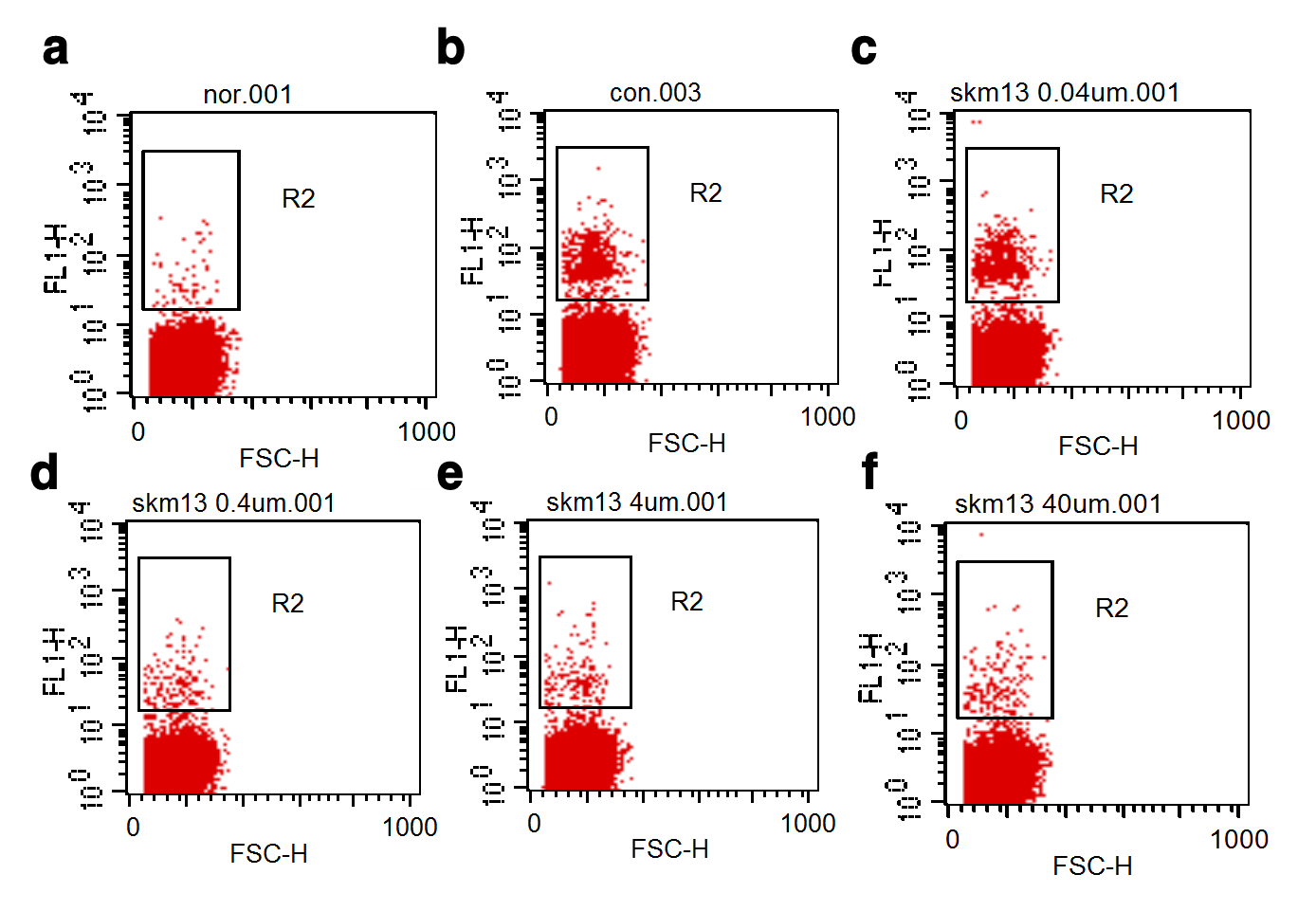
**

**Fig. S2. FACS analysis for IC_50_ of SKM13 in *P.falciparum* (3D7).** Each small rectangular square indicates the positive fluorescence labeling. **a**, uninfected RBC; **b**, infected RBC+DMSO; **c**, infected RBC+ 0.04 μM of SKM13; **d**, infected RBC+ 0.4 μM of SKM13; **e**, infected RBC+ 4 μM of SKM13; **f**, infected RBC+ 40 μM of SKM13.

**
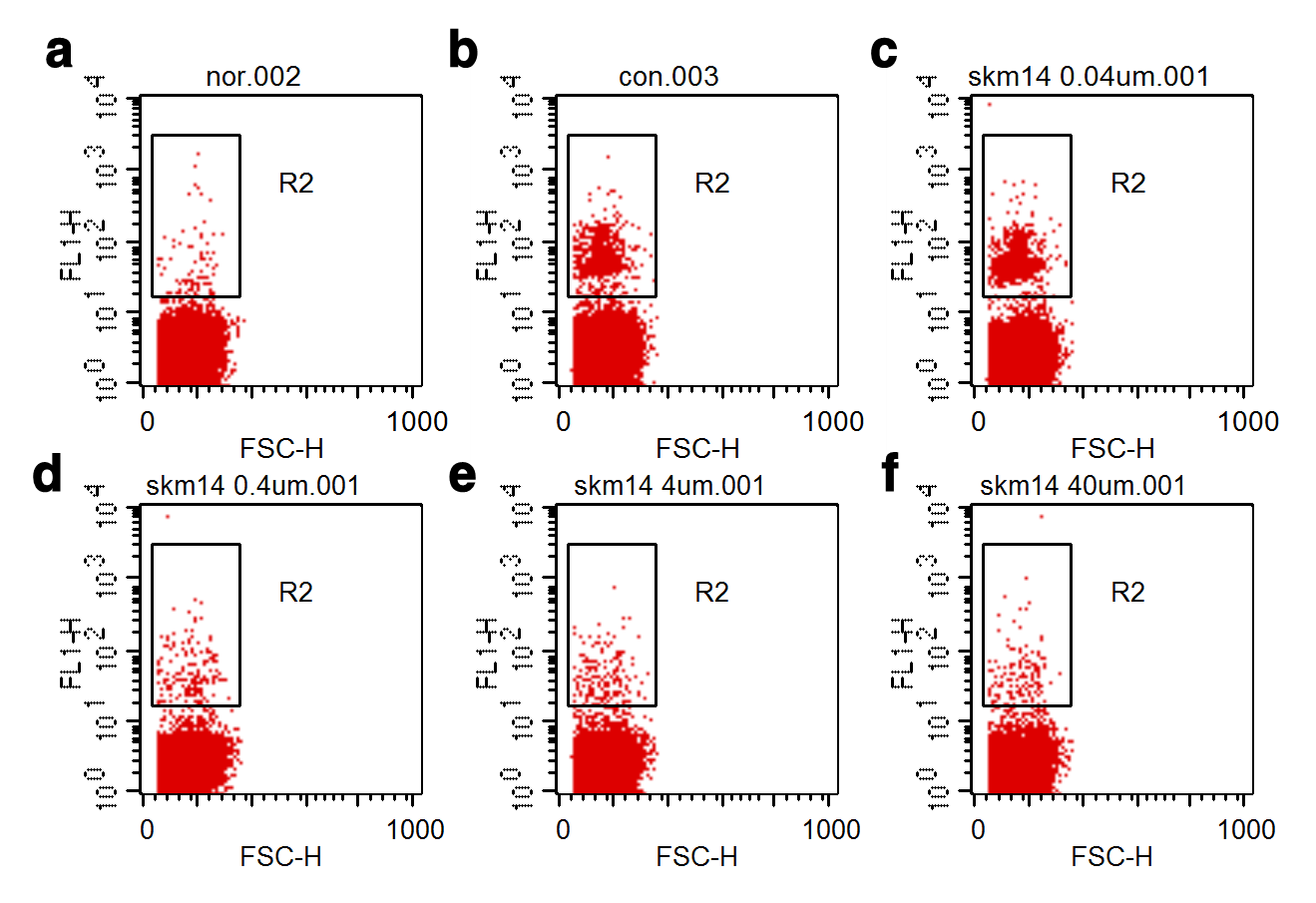
**

**Fig. S3. FACS analysis for IC_50_ of SKM14 in *P.falciparum* (3D7).** Each small rectangular square indicates the positive fluorescence labeling. **a**, uninfected RBC; **b**, infected RBC+DMSO; **c**, infected RBC+ 0.04 μM of SKM14; **d**, infected RBC+ 0.4 μM of SKM14; **e**, infected RBC+ 4 μM of SKM14; **f**, infected RBC+ 40 μM of SKM14.

**
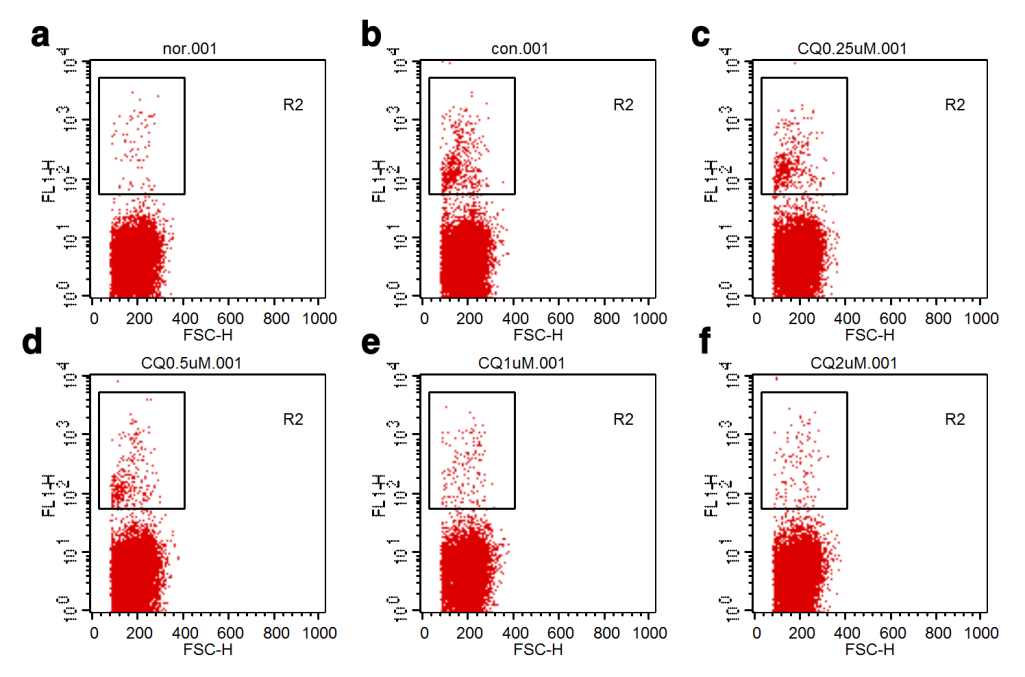
**

**Fig. S4. FACS analysis for IC_50_ of CQ in *P.falciparum* (FCR3).** Each small rectangular square indicates the positive fluorescence labeling. **a**, uninfected RBC; **b**, infected RBC+DMSO; **c**, infected RBC+ 0.25 μM of CQ; **d**, infected RBC+ 0.5 μM of CQ; **e**, infected RBC+ 1 μM of CQ; **f**, infected RBC+ 2 μM of CQ.

**
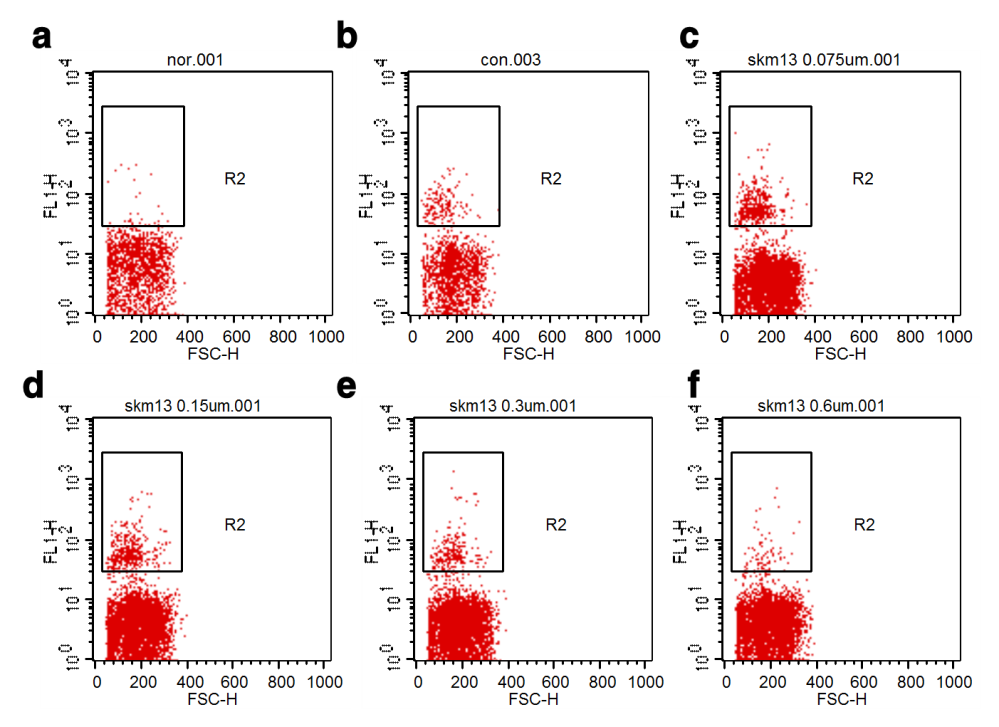
**

**Fig. S5. FACS analysis for IC_50_ of SKM13 in *P.falciparum* (FCR3).** Each small rectangular square indicates the positive fluorescence labeling. **a**, uninfected RBC; **b**, infected RBC+DMSO; **c**, infected RBC+ 0.075 μM of SKM13; **d**, infected RBC+ 0.15 μM of SKM13; **e**, infected RBC+0.3 μM of SKM13; **f**, infected RBC+ 0.6 μM of SKM13.


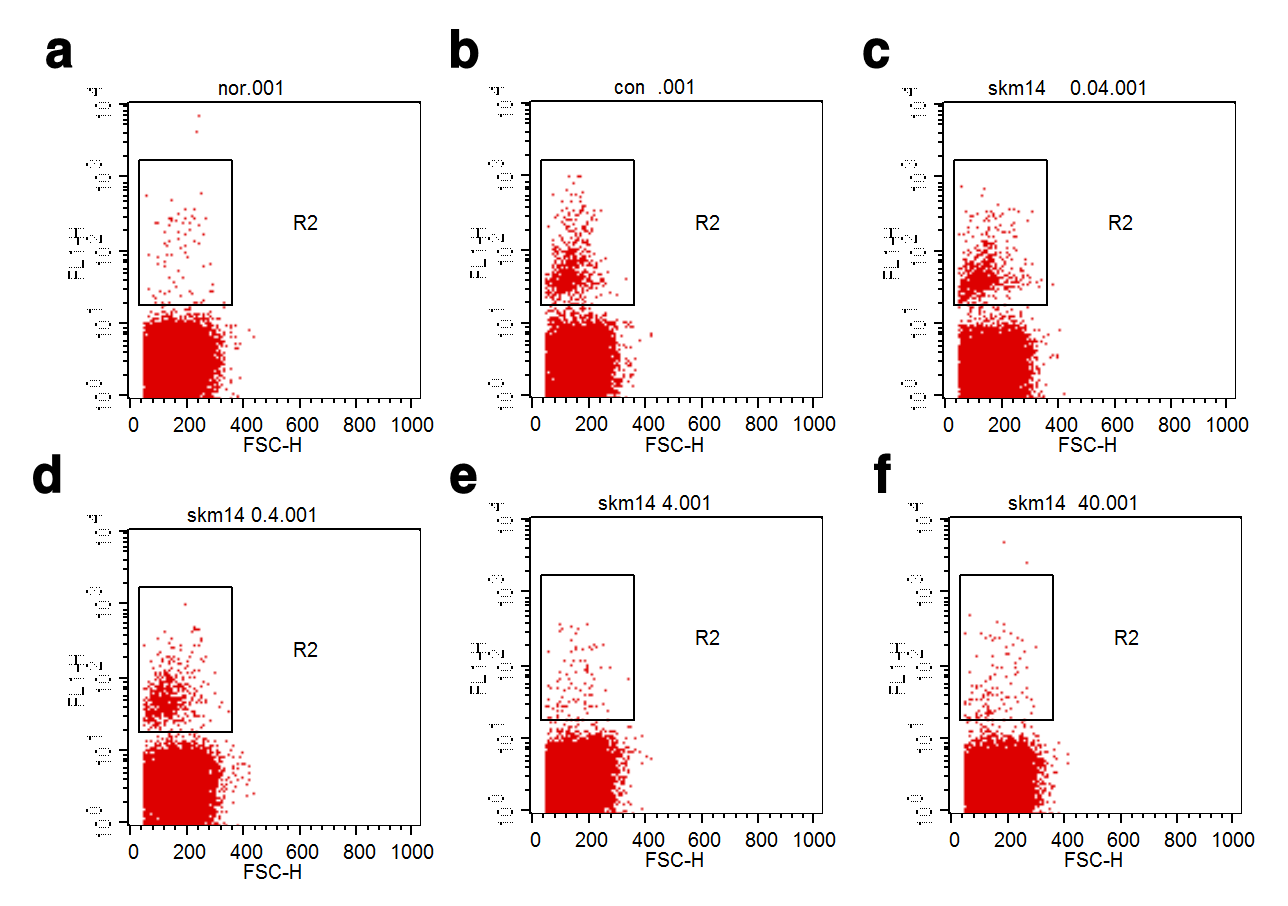


**Fig. S6. FACS analysis for IC_50_ of SKM14 in *P.falciparum* (FCR3).** Each small rectangular square indicates the positive fluorescence labeling. **a**, uninfected RBC; **b**, infected RBC+DMSO; **c**, infected RBC+ 0.04 μM of SKM14; **d**, infected RBC+ 0.4 μM of SKM14; **e**, infected RBC+ 4 μM of SKM14; **f**, infected RBC+ 40 μM of SKM14.
